# Matched related transplantation versus immunosuppressive therapy plus eltrombopag for first-line treatment of severe aplastic anemia: a multicenter, prospective study

**DOI:** 10.1186/s13045-022-01324-1

**Published:** 2022-08-12

**Authors:** Limin Liu, Meiqing Lei, Rong Fu, Bing Han, Xin Zhao, Rongrong Liu, Yanming Zhang, Wenjing Jiao, Miao Miao, Fengkui Zhang, Liansheng Zhang, Depei Wu

**Affiliations:** 1 National Clinical Research Center for Hematologic Diseases, Jiangsu Institute of Hematology, The First Affiliated Hospital of Soochow University, Institute of Blood and Marrow Transplantation of Soochow University, 188 Shizi Street, Suzhou, 215006 Jiangsu Province China; 2 Department of Hematology, Haikou Municipal People’s Hospital, Affiliated Haikou Hospital Xiangya School of Medicine Central South University, Haikou, Hainan Province China; 3 Department of Hematology, General Hospital, Tianjin Medical University, Tianjin, China; 4 Department of Hematology, Peking Union Medical College Hospital, Chinese Academy of Medical Sciences and Peking Union Medical College, Beijing, China; 5 State Key Laboratory of Experimental Hematology, National Clinical Research Center for Blood, Diseases, Anemia Therapeutic Center, Institute of Hematology & Blood Diseases Hospital, Chinese Academy of Medical Sciences & Peking Union Medical College, Tianjin, China; 6 Department of Hematology, The First Affiliated Hospital of Guangxi Medical University, Nanning, China; 7 Department of Hematology, The Affiliated Huai’an Hospital of Xuzhou Medical University and The Second People’s Hospital of Huai’an, Huai’an, Jiangsu Province China; 8 Department of Hematology, Xian Yang Central Hospital, Xianyang, Shanxi Province China; 9 Department of Hematology, The Second Hospital of Lanzhou University, Lanzhou, 730000 Gansu Province China

**Keywords:** Matched related transplantation, Immunosuppressive therapy, Eltrombopag, Severe aplastic anemia

## Abstract

This study prospectively compared the efficacy and safety between matched related donor-hematopoietic stem cell transplantation (MRD-HSCT) (*n* = 108) and immunosuppressive therapy (IST) plus eltrombopag (EPAG) (IST + EPAG) (*n* = 104) to determine whether MRD-HSCT was still superior as a front-line treatment for patients with severe aplastic anemia (SAA). Compared with IST + EPAG group, patients in the MRD-HSCT achieved faster transfusion independence, absolute neutrophil count ≥ 1.0 × 10^9^/L (*P* < 0.05), as well as high percentage of normal blood routine at 6-month (86.5% vs. 23.7%, *P* < 0.001). In the MRD-HSCT and IST + EPAG groups, 3-year overall survival (OS) was 84.2 ± 3.5% and 89.7 ± 3.1% (*P* = 0.164), whereas 3-year failure-free survival (FFS) was 81.4 ± 4.0% and 59.1 ± 4.9% (*P* = 0.002), respectively. Subgroup analysis indicated that the FFS of the MRD-HSCT was superior to that of the IST + EPAG among patients aged < 40 years old (81.0 ± 4.6% vs. 63.7 ± 6.5%, *P* = 0.033), and among patients with vSAA (86.1 ± 5.9% vs. 54.9 ± 7.9%, *P* = 0.003), while the 3-year OS of the IST + EPAG was higher than that of the MRD-HSCT among the patient aged ≥ 40 years old (100.0 ± 0.0% vs. 77.8 ± 9.8%, *P* = 0.036). Multivariate analysis showed that first-line MRD-HSCT treatment was associated favorably with normal blood results at 6-month and FFS (*P* < 0.05). These outcomes suggest that MRD-HSCT remains the preferred first-line option for SAA patients aged < 40 years old or with vSAA even in the era of EPAG.

**Supplementary Information:**

The online version contains supplementary material available at 10.1186/s13045-022-01324-1.

**To the editor**

Allogeneic hematopoietic stem cell transplantation (allo-HSCT) and immunosuppressive therapy (IST) are the two main treatment strategies for newly diagnosed severe aplastic anemia (SAA) [1]. In spite of comparable overall survival (OS) between matched related donor-HSCT (MRD-HSCT) and IST, failure-free survival (FFS) has been reported to often be better in the former than the latter [2, 3], mainly because of a low rate of complete response (CR) and relatively high rate of relapse and clonal evolution after IST treatment [4]. In recent years, the addition of eltrombopag (EPAG), an oral synthetic small-molecule thrombopoietin receptor agonist, to standard IST has been shown to improve the speed and depth of a hematological response in patients with SAA, without additional toxic effects [5, 6]. Until recently, a study comparing MRD-HSCT and IST + EPAG as first-line treatment suggested MRD-HSCT achieved a better OS in patients with very SAA (vSAA) and a higher FFS in all SAA patients. However, this study had some limitations, such as a small sample size and imbalanced characteristics, which may have reduced the reliability of the comparison outcome to some extent [7]. Therefore, it remains necessary to investigate whether the improvements associated with IST + EPAG were comparable to those of MRD-HSCT in SAA using a larger and well-designed study.

This multicenter prospective study compared the efficacy and safety of MRD-HSCT and IST + EPAG as front-line therapy in contemporaneous SAA patients. Of the 216 patients enrolled in the study between January 2016 and February 2021, 108 received MRD-HSCT treatment, with the implementation process of the treatment being the same as that used in our previous study [8]. All the patients were analyzed. In the 108 patients who received IST + EPAG treatment, standard IST was administered as described in our previous study [9], while EPAG was initiated on day 1 at a dose of 50 mg per day and then increased by 25 mg every 2 weeks until a maximum of 150 mg or a dose resulting in a hematological response. Finally, only 104 patients administered EPAG for at least three months were eligible for analysis. Patients/methods and results are shown in Additional file 1. The characteristics of the patients in the two groups are shown in Table 1. The median age was lower in the MRD-HSCT group than that in the IST + EPAG group (*P* = 0.024). The median interval from diagnosis to treatment was longer in the MRD-HSCT group than that in the IST + EPAG group (*P* = 0.011) (Table 1). In the MRD-HSCT group, all the patients achieved myeloid engraftment, complete donor chimerism, engraftment time, and secondary GF (Table 1). In addition, the aGVHD/cGVHD incidence was acceptable (Fig. 1A, B), which was similar to the results of a previous study [10]. The adverse effects observed for EPAG included hyperbilirubinemia, liver enzyme elevation, and dyspepsia, all of which were ameliorated quickly by intensive supportive treatment, with the overall tolerability acceptable and consistent with the previously reported safety profile of EPAG [5, 11].

**Table 1:** Characteristics of patient and donor (graft) and clinical outcomes between the two groups

Variables	MRD-HSCT (*n* = 108)	IST + EPAG (*n* = 104)	*P*
Median age, yr (range)	29 (6–56)	34.5 (4–69)	**0.024**
Age, no. (%)			< **0.001**
< 20 yr	17 (15.7)	24 (23.1)	
20–40 yr	67 (62.0)	32 (30.8)	
≥ 40 yr	24 (22.2)	48 (46.2)	
Sex, no. (%)			0.767
Male	57 (52.8)	57 (54.8)	
Female	51 (47.2)	47 (45.2)	
Disease status, no. (%)			0.831
SAA	68 (66.7)	64 (61.5)	
vSAA	40 (33.3)	40 (38.5)	
With PNH clone, no. (%)	26 (24.1)	19 (10.2)	0.301
ECOG score, median (range)	1 (0–2)	1 (0–2)	0.537
Median time from diagnosis to treatment, mth (range)	3 (1.0–200)	2 (0.5–240)	**0.011**
Median time to an ANC ≥ 1.0 × 10^9^/L, d (range)	15 (11–35)	30 (4–58)	**0.002**
Median time to transfusion independence for RBCs, d (range)	22 (13–32)	63 (6–302)	< **0.001**
Median time to transfusion independence for platelets, d (range)	12 (8–52)	52 (11–287)	< **0.001**
Normal blood routine at 6-mth, no. (%)	83 (86.5)	23 (23.7)	< **0.001**
Early death, no (%)	7 (6.5)	2 (1.9)	0.192
Secondary clonal disease, no (%)	1 (0.9)	2 (2.9)	0.587
Relapsed, no (%)	0 (0.0)	1 (0.9)	–
Alternative donor transplantation, no (%)	0 (0.0)	10 (9.6)	–
TRM, no (%)	17 (15.7)	10 (9.6)	0.181
Secondary GF, no (% of TRM)	1 (5.9)	–	
aGVHD, no (% of TRM)	1 (5.9)	–	
cGVHD, no (% of TRM)	1 (5.9)	–	
TMA, no (% of TRM)	2 (11.8)	–	
Poor graft function, no (% of TRM)	1 (5.9)	–	
Infection, no (% of TRM)	9 (52.8)	5 (50.0)	
Intracranial hemorrhage, no (% of TRM)	1 (5.9)	2 (20.0)	
Heart failure, no (% of TRM)	–	1 (10.0)	
Other, no (% of TRM)	1 (5.9)	2 (20.0)	
Median follow-up time among living patients, mth (range)	31.5 (13.0–69.0)	30.5 (14.0–66.0)	0.589
Conditioning regimen	Flu + CY + ATG	rATG (pALG) + CsA + EPAG	
Donor median age, yr (range)	30 (10–55)	–	
Donor sex, no. (%)		–	
Male	54 (50.0)	–	
Female	54 (50.0)	–	
Blood types of donor to recipient, no. (%)			
Matched	65 (60.2)	–	
Major mismatched	15 (13.9)	–	
Minor mismatched	19 (17.6)	–	
Major and minor mismatched	9 (8.3)	–	
Source of graft, no. (%)			
BM	4 (3.7)	–	
PB	30 (27.8)	–	
BM + PB	74 (68.5)	–	
Median MNC, × 10^8^/kg (range)	11.6 (3.2–24.4)	–	
Median CD34^+^ cells, × 10^6^/kg (range)	3.7 (1.1–8.6)	–	
Median time to ANC > 0.5 × 10^9^/L, d (range)	11 (7–21)	–	
Median time to PLT > 20.0 × 10^9^/L, d (range)	12 (8–52)	–	
Primary GF, no. (%)	0 (0.0)	–	
Secondary GF, no. (%)	2 (1.9)	–	
GF of platelet, no. (%)	3 (2.9)	–	
Delayed platelet recovery, no. (%)	4 (3.8)	–	
Poor graft function, no. (%)	1 (0.9)	–	
Adverse events of attributed to EPAG, no. (%)			
Skin (maculopapular and/or rash pruritus)	–	3 (2.9)	
Abdominal pain	–	2 (1.9)	
Joint pain	–	2 (1.9)	
Liver test abnormality			
Increased aminotransferase level	–	42 (40.4)	
Increased blood bilirubin level	–	19 (18.3)	

The bold values were statistically significant

*MRD-HSCT* matched related donor hematopoietic stem cell transplantation, *IST* immunosuppressive therapy, *EPAG* eltrombopag, *SAA* severe aplastic anemia, *vSAA* very SAA, *PNH* paroxysmal nocturnal hemoglobinuria, *ECOG* Eastern Cooperative Oncology Group Scale, *BM* bone marrow, *PB* peripheral blood, *MNC* mononuclear cell, *ANC* absolute neutrophil count, *PLT* platelet, *GF* graft failure, *TRM* treatment related mortality

**Fig. 1:**
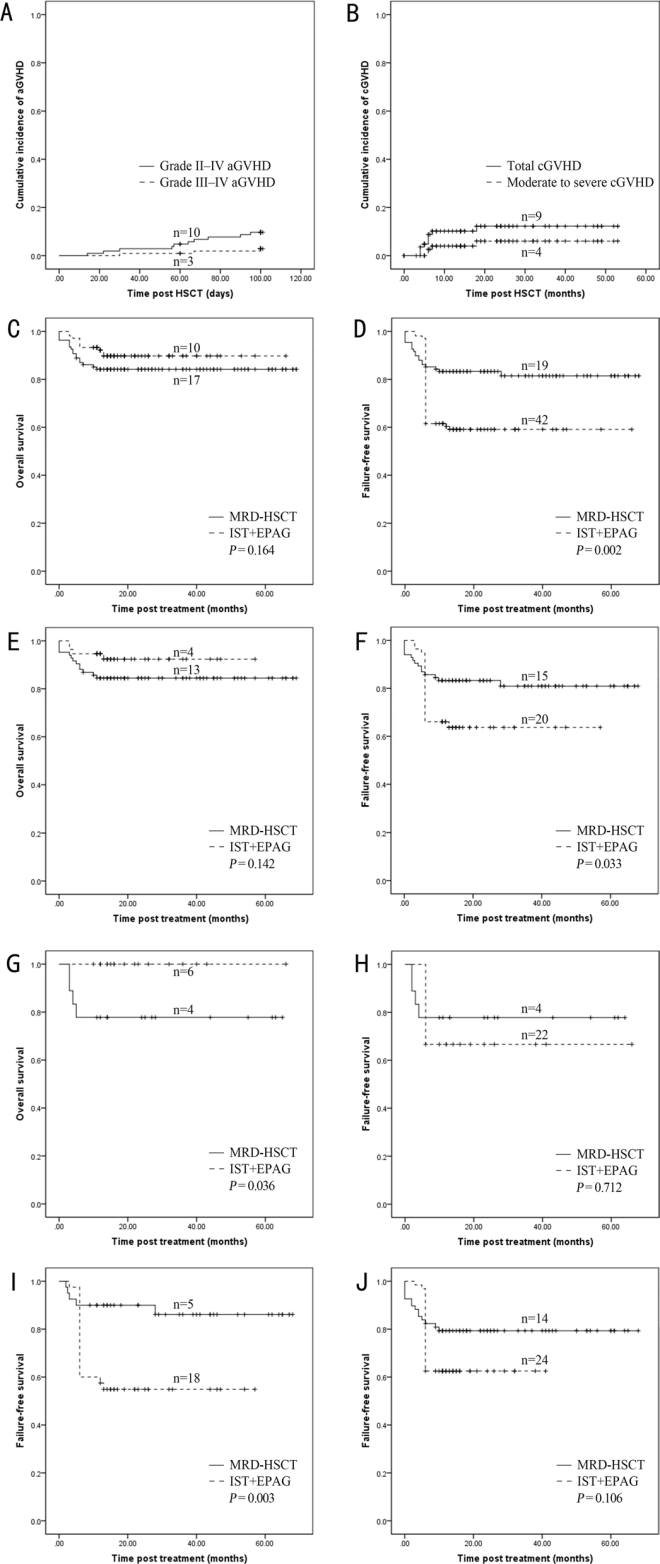
GVHD after transplantation and survival after treatment with MRD-HSCT or IST + EPAG (including subgroups). **A** Grade II–IV acute GVHD (aGVHD) and Grade III–IV aGVHD after MRD-HSCT. **B** Total chronic GVHD (cGVHD) and moderate-to-severe cGVHD after MRD-HSCT. **C** OS between MRD-HSCT and IST + EPAG groups as a whole. **D** FFS between MRD-HSCT and IST + EPAG groups as a whole. **E** OS between MRD-HSCT and IST + EPAG subgroups for patients with aged < 40 years. **F** FFS between MRD-HSCT and IST + EPAG subgroups for patients aged < 40 years. **G** OS between MRD-HSCT and IST + EPAG subgroups for patients with age ≥ 40 years. **H** FFS between MRD-HSCT and IST + EPAG subgroups for patients with age ≥ 40 years. **I** FFS between MRD-HSCT and IST + EPAG subgroups for patients with vSAA. **J** FFS between MRD-HSCT and IST + EPAG subgroups for patients with SAA

At 6 months post-treatment, the time taken to achieve transfusion independence, absolute neutrophil count (ANC) ≥ l × 10^9^/L, and subsequent normal blood results all favored the MRD-HSCT group compared with that observed in the IST + EPAG group (Table 1). Further details about the IST + EPAG response rate at 3 and 6 months are shown in Additional file 2. These results were similar to those of another study [7]. Multivariate analysis showed first-line MRD-HSCT was the only treatment associated with normal blood results six months after treatment (*P* < 0.001). Next, we performed a survival comparison between the MRD-HSCT and IST + EPAG groups and each age subgroup. For the total population or patients aged < 40 years old, although there was no difference in OS between the MRD-HSCT and IST + EPAG groups, a significant higher FFS rate was found in the former than that in the latter (Fig. 1C–F). For patients aged ≥ 40 years old after propensity score matching (the propensity score was calculated based on a multivariate logistic regression model, which took patient age, sex, and disease status between the two groups as covariates), there was a comparable FFS rate, with a higher OS rate in the IST + EPAG subgroup than in the MRD-HSCT subgroup (Fig. 1G, H). For patients aged < 20 and 20–39 years old, there were no significant differences in OS or FFS between the MRD-HSCT and IST + EPAG groups (Additional file 3). Furthermore, FFS in the IST + EPAG group was still inferior to that of the MRD-HSCT group in patients with SAA. It is remarkable that FFS in the IST + EPAG group was significantly inferior to that of the MRD-HSCT group in patients with vSAA (Fig. 1I, J), which is consistent with the outcomes of a recent study [7].

In the current study, the FFS rate of approximately 60% in the IST + EPAG group was mainly due to the high none response rate of up to 30% at 6 months (Table 1), which was similar to that reported by another study [6]. Multivariate analysis showed that first-line MRD-HSCT and < 4 months between diagnosis and treatment were two favorable factors for FFS for the total population (*P* = 0.001 and *P* = 0.008, respectively, Additional file 4).

Notably, the survival outcomes in the IST + EPAG group may be due partly to patients who did not respond to initial therapy who subsequently received an allo-HSCT from alternative donors. Even so, our data still support that MRD-HSCT should be recommended in SAA patients as first-line treatment rather than IST + EPAG, especially for those younger than 40 years old or with vSAA, while transplants for patients older than 40 years carried a significant risk of mortality.

## Supplementary Information

**Additional file 1.** Patients/methods and results.

**Additional file 2: Table S1.** Response rates at 6-month in IST + EPAG whole group or subgroup.

**Additional file 3: Fig. S1.** Survival after treatment with MRD-HSCT or IST + EPAG for patients with further age stratification. (**A**) OS between MRD-HSCT and IST + EPAG subgroups for patients with aged < 20 years. (**B**) FFS between MRD-HSCT and IST + EPAG subgroups for patients age < 20 years. (**C**) OS between MRD-HSCT and IST + EPAG subgroups for patients with age 20–39 years. (**D**) FFS between MRD-HSCT and IST + EPAG subgroups for patients with age 20–39 years.

**Additional file 4: Table S2.** Multivariate analysis of factors associated with favorable outcomes.

## Data Availability

The datasets used and/or analyzed during the present study are available from the corresponding author on reasonable request.

## Abbreviations

aGVHD: Acute graft-versus-host disease
allo-HSCT: Allogeneic hematopoietic stem cell transplantation
ANC: Absolute neutrophil count
BM: Bone marrow
BU: Busulfan
cGVHD: Chronic graft-versus-host disease
CsA: Cyclosporin A
CY: Cyclophosphamide
EPAG: Eltrombopag
ECOG: Eastern Cooperative Oncology Group
FFS: Failure-free survival
Flu: Fludarabine
GF: Graft failure
HLA: Human leukocyte antigen
IST: Immunosuppressive therapy
MNCs: Mononuclear cells
MTX: Methotrexate
MRD: Matched related donor
MMF: Mycophenolate mofetil
OS: Overall survival
PLT: Platelet
PNH: Paroxysmal nocturnal hemoglobinuria
PBSCs: Peripheral blood stem cells
pALG: Pig antilymphocyte immunoglobulin
rATG: Rabbit antithymocyte immunoglobulin
SAA: Severe aplastic anemia
TRM: Transplantation-related mortality
