# Supplementary material for: Matched related transplantation versus immunosuppressive therapy plus eltrombopag for first-line treatment of severe aplastic anemia: a multicenter, prospective study
Source: J Hematol Oncol. 2022 Aug 12;15:105. doi: 10.1186/s13045-022-01324-1 (PMC9373485; doi:10.1186/s13045-022-01324-1)
Supplement: Supplementary file 1 — Additional file 1. Patients/methods and results. [file 13045_2022_1324_MOESM1_ESM.docx]

**PATIENTS AND METHODS**

**Patients**

**Study design and participants**

The study was a prospective, multicenter design that included 216 SAA patients from nine participating institutions in China, enrolled between January 2016 and February 2021. According to current therapeutic algorithms, allo-HSCT is recommended as a first-line intervention in young SAA patients aged < 40 years with a matched donor, while allo-HSCT can be used in some older SAA patients (40–60 years) with a matched donor who has volunteered to participate in transplantation. IST + EPAG is recommended as first-line treatment for young patients who do not have a MRD and patients older than 40 years. This study was approved by the local institutional review board. All of the patients or their guardians signed a written, informed consent form in accordance with the Declaration of Helsinki. Patients were followed-up until the end of the study evaluation period of February 28th, 2022.

The inclusion criteria of patients were (1) a diagnosis of acquired SAA, including very SAA (vSAA), as described previously; (2) transfusion-dependent. The exclusion criteria of SAA or vSAA included: (1) secondary factors such as a history of viral infection or exposure to drugs or toxic agents; (2) congenital bone marrow failure; (3) patients with an uncontrolled or active infection; (4) patients with severe liver, kidney, lung, or heart disease; (5) patients with a relatively poor performance status (ECOG > 2 points); and (6) pregnant patients.

Expression of CD55 and CD59 in peripheral blood cells and cytogenetic analyses from bone marrow were also screened routinely in all the patients

**Treatment protocol of MRD-HSCT**

Details of the conditioning regimen, stem cell mobilization, collection and infusion, graft-versus-host disease (GVHD) prophylaxis and treatment strategy, supportive care, and post-transplantation surveillance were the same as that used in our previous studies.

**Treatment regimen for IST+ EPAG**

In the IST+EPAG group, standard IST consisted of rabbit ATG (rATG) 3–4 mg/kg/day or porcine antihuman lymphocyte immunoglobulin (pALG) 20–30 mg/kg/day from days 1 to 5, and oral CsA (3–5 mg/kg/day) over the same time period, with adjustment of the dose to achieve a whole blood trough concentration of 200–250 ng/mL in adults and 150–200 ng/ml in children. The effective dosage of CsA was maintained for at least 12 months, followed by a slow taper until discontinuation (at total course of at least two years). EPAG was initiated on day 1 at a dose of 50 mg per day and then increased by 25 mg every 2 weeks until a maximum of 150 mg or a dose resulting in a hematological response. Patients who had been administered EPAG for at least three months were eligible for the final analysis.

During the entire treatment, short courses of G-CSF (5 μg/kg/day) were administered when the absolute neutrophil count (ANC) was < 0.5 × 10^9^/L and discontinued when the ANC was > 1.0 × 10^9^/L. Prophylactic antifungal (voriconazole 200 mg/12 h PO or micafungin 100–300 mg/day i.v.) and antiviral (acyclovir 200 mg/8 h PO) agents were administered during the immunosuppressive period as described in our previous study.

**Definitions**

Neutrophil engraftment was defined as the first day of an ANC > 0.5 × 10^9^/L for three consecutive days. Platelet engraftment was defined as the first day of a platelet count > 20 × 10^9^/L for seven consecutive days without transfusion support. Primary graft failure (GF) was defined as failure to achieve neutrophil engraftment until longer than 28 days. Secondary GF was defined as recurrent pancytopenia with an ANC < 0.5 × 10^9^/L after a prior successful engraftment, and mixed chimerism (MC) as 5% to 95% for both myeloid and lymphoid lineages from the donor-type(24). Delayed platelet recovery was defined as no platelet engraftment until longer than 30 days. Poor graft function was defined as persistent cytopenia in at least two lineages (platelet < 20 × 10^9^/L, neutrophil count < 0.5 × 10^9^/L, or hemoglobin level < 70 g/L) and/or requiring a transfusion after longer than 28 days, and full donor chimerism without relapse or severe GVHD.

In the IST+EPAG group, CR was defined as a normal hemoglobin level for gender, an ANC > 1.5 × 10^9^/L, and a platelet count > 150 × 10^9^/L. A partial response (PR) was defined as transfusion independent and no longer meeting the criteria for SAA/vSAA. An absence of response was defined as still meeting the severe disease criteria.

Early mortality was defined as death within 60 days after HSCT. Transplantation-related mortality (TRM) was defined as death related to the transplantation and not the relapse of SAA. FFS was defined as survival with treatment response. Death, NR by 6 months, disease progression

requiring clinical intervention, clonal evolution, and relapse were considered treatment failures for IST; death, primary or secondary GF, graft rejection, secondary malignancy, and relapse were considered treatment failures for HSCT.

**Statistical analysis**

The statistical analyses were conducted using data available from the start of treatment to the final date of follow-up, February 28th, 2022. Patient characteristics were compared using the chi-square test and the nonparametric test for continuous variables. Cumulative incidences of GVHD were estimated using the competing risk model, with death as the competing event. The probabilities of OS and FFS were performed using the Kaplan–Meier method and compared using the log-rank test. To reduce the influence of potential confounders in patients aged ≥ 40 years, propensity score matching was performed using Rv 2.15.0 software. The propensity score was calculated based on a multivariate logistic regression model. In this model, patient age, sex, and disease status between the two groups were used as covariates. Patients in the MRD-HSCT group were matched to those in the IST+EPAG group using 1:1 nearest neighbor matching with a caliper width of 0.2. The multivariate analyses were performed using a Cox proportional hazard regression model. The statistical analyses were performed using SPSS version 22.0 (SPSS, Chicago, IL, U.S.). All *P* values were two-sided and the results considered statistically significant when *P* < 0.05.

**RESULTS**

**Patient characteristics**

A total of 216 patients were enrolled in the study. Of these, 108 patients received MRD-HSCT treatment and 104 patients received IST+EPAG treatment (4 patients in IST+EPAG group was excluded for administering EPAG less than 3 months). Differences in the characteristics of the patients in the two groups are shown in Table 1. There was no difference in gender, disease status, PNH clone, and ECOG score (all *P <* 0.05). The median age was lower in the MRD-HSCT group than in the IST+EPAG group (*P* = 0.024). Accordingly, the proportion of patients younger than 40 years in the MRD-HSCT group was higher than that in the IST+EPAG group. The median interval from diagnosis to treatment was longer in the MRD-HSCT group than that in the IST+EPAG group (*P* = 0.011).

**Outcomes of MRD-HSCT**

In the MRD-HSCT group, 104 of the 108 patients survived for longer than 28 days, the median mononuclear count (MNC) of the grafts was 11.6 (range, 3.2–24.4) × 10^8^/kg, and the CD34^+^ cell count was 3.7 (range, 1.1–8.6) × 10^6^/kg. All the 104 evaluable patients achieved initial full donor chimerism. The median time to neutrophil engraftment was 11 days (range, 7–21), while the median time to platelet engraftment was 12 days (range, 8–52) (Table 1). The median time taken for ANC ≥ l × 10^9^/L was 15 days (range, 13–35). The median time to transfusion independence was 22 days (range, 13–32) for red cells and 12 days (range, 8–52) for platelets. As shown in Table 1, 6 months after MRD-HSCT 83 patients of the 96 evaluable patients (86.5%) had achieved normal blood results. At the time of the last follow-up, one patient experienced secondary GF with complete recipient-type nine months after transplantation and soon died. Another patient experienced secondary GF with MC 27 months after transplantation and soon became independent of transfusion by adjustment of the CsA dose and administration of G-CSF and EPAG. 3 patients experienced GF of platelets, 4 patients experienced delayed platelet engraftment, and 1 patient experienced poor graft function (Table 1).

The cumulative incidence on day +100 for grades II–IV acute GVHD (aGVHD) was 10.7 ± 2.9%, and for grades III–IV aGVHD was 2.9 ± 1.6% (Figure 1A). The cumulative incidence of chronic GVHD (cGVHD) was 10.2 ± 3.4% and the cumulative incidence of moderate–severe cGVHD was 6.0 ± 3.0% (Figure 1B).

Multivariate analysis showed that the choice of first-line MRD-HSCT was the only favorable factor for achieving normal blood results 6 months after treatment (*P* < 0.001) (Additional file 4).

**Outcomes of IST+EPAG and EPAG duration/withdrawal**

6 months after IST + EPAG treatment, 7 patients were not evaluable because of either death (n = 6) or because they had undergone allo-HSCT from alternative donors within 6 months (n = 1). Of the 97 patients evaluable for a response, 23 patients (23.7%) achieved a CR, 43 patients (44.3%) achieved a PR, and 31 patients (32.0%) achieved a NR. In the IST+EPAG group, 52 patients received r-ATG and 52 patients received p-ALG, and the response rates for these two subgroup were added into additional file 2. 6 months after treatment, the percentage of patients with normal blood routine was significantly higher in the MRD-HSCT group than in the IST+EPAG group (86.5% vs. 23.5%, *P* < 0.001) (Table 1). In the patients who responded, the median time taken for ANC to reach ≥ l × 10^9^/L for three consecutive days was 30 days (range, 4–58). The median time required to discontinue transfusion of red blood cells and platelets was 63 days (range, 2–289) and 56 days (range, 0–295) respectively. The time for ANC to reach ≥ l × 10^9^/L and independence from transfusion in the IST+EPAG group was significantly longer than that observed in the MRD-HSCT group (*P* all < 0.05) (Table 1).

Overall, patients received EPAG for a median of 6.5 months (4.1–23.2) at a median dose of 100 mg/day (range, 75–150). 45 patients were still taking EPAG orally until the last follow-up.

**TRM, relapse, secondary clonal evolution**

During the follow-up period, TRM in the MRD-HSCT and IST+EPAG groups was 15.7% and 9.6%, respectively (*P =* 0.181). In the MRD-HSCT group, 9 patients (52.8%) died from an infection, 2 patients (11.8%) died from GVHD (1 aGVHD and 1 cGVHD), and 1 patient (5.9%) died from intracranial hemorrhage. In the IST+EPAG group, 5 patients (50.0%) died from infection and 2 patients (20.0%) died from intracranial hemorrhage. Additional details of transplant-related events are shown in Table 1. No patient in the MRD-HSCT group experienced a relapse, while one (1.0%) responder in the IST+EPAG group experienced a relapse. In the MRD-HSCT group 1 patient (0.9%) developed karyotypic evolution (monosomy 7), and in the IST+EPAG group 3 (2.9%) responders developed karyotypic abnormalities (1 paroxysmal nocturnal hemoglobinuria, 1 monosomy 7 and 1 del [13q]) (*P* = 0.587) (Table 1). Up to February 28th, 2022, 10 patients in the IST+EPAG group received either salvage HSCT from matched unrelated donor (n = 1) or haploidentical family donors (n = 8) because of NR, or unrelated cord blood (n = 1) because of relapse. No patient in the MRD-HSCT group received a second allo-HSCT.

**Survival**

The estimated OS at 3-year was 84.2 ± 3.5% in the MRD-HSCT group and 89.7 ± 3.1% in the IST+EPAG group (*P* = 0.164) (Figs 1C), while the estimated FFS at 3-year was 81.4 ± 4.0% in the MRD-HSCT group and 59.1 ± 4.9% in the IST+EPAG group (*P* = 0.002) (Figs 1D). For patients aged < 40 years, the estimated OS at 3-year was 84.5 ± 4.0% in the MRD-HSCT subgroup and 92.4 ± 3.7% in the IST+EPAG subgroup (*P* = 0.142) (Figs 2E), while the estimated FFS at 3-year was 81.0 ± 4.6% in the MRD-HSCT subgroup and 63.7 ± 6.5% in the IST+EPAG subgroup (*P* = 0.033) (Figs 2F). After propensity score matching (1:1) in patients aged ≥ 40 years, 18 pairs of patients were created. In the matched-pair patients, the estimated OS at 3-year was 77.8 ± 9.8% in the MRD-HSCT subgroup and 100.0 ± 0.0% in the IST+EPAG subgroup (*P* = 0.036) (Figs 2G), while the estimated FFS at 3-year was 77.8 ± 9.8% in the MRD-HSCT subgroup and 66.7 ± 7.3% in the IST+EPAG subgroup (*P* = 0.712) (Figs 1H).

For the patients aged < 20 and 20-39 years old, there was not significantly different in OS and FFS between the MRD-HSCT and IST+EPAG groups (Additional file 3). Considering that reticulocyte and neutrophil counts were two predictors of hematologic response of aplastic anemia to ATG, non-vSAA patients maybe display higher FFS than vSAA patients when treated with IST+EPAG. In present study, FFS of IST+EPAG was still inferior to that of MRD-HSCT among patients with SAA, It is remarkable that FFS of IST+EPAG was significantly inferior to that of MRD-HSCT among patients with vSAA ( (Figs 1I and 1J).

Multivariate analysis showed that there was no favorable factor for OS (*P* all > 0.05), although FFS was significantly higher in the entire population receiving MRD-HSCT and in those who received MRD-HSCT or IST+EPAG treatment less than 4 months from the time of diagnosis (*P* = 0.001 and *P* = 0.008, respectively, Additional file 4).
