# Supplementary material for: Matched related transplantation versus immunosuppressive therapy plus eltrombopag for first-line treatment of severe aplastic anemia: a multicenter, prospective study
Source: J Hematol Oncol. 2022 Aug 12;15:105. doi: 10.1186/s13045-022-01324-1 (PMC9373485; doi:10.1186/s13045-022-01324-1)
Supplement: Supplementary file 2 — Additional file 2: Table S1. Response rates at 6-month in IST + EPAG whole group or subgroup. [file 13045_2022_1324_MOESM2_ESM.doc]

**Table S 1. Response rates at 6-month in IST+EPAG whole group or subgroup**

| **Response rates** | **IST+EPAG (n = 104)** | **rATG**  **(n = 52)** | **pALG**  **(n = 52)** |
| --- | --- | --- | --- |
| Complete response, no (% of evaluable patients) | 23 (23.7) | 11 (23.4) | 12 (24.0) |
| Partial response, no (% of evaluable patients) | 43 (44.3) | 25 (53.2) | 18 (36.0) |
| None response, no (% of evaluable patients) | 31 (32.0) | 11 (23.4) | 20 (40.0) |
| Alternative donor transplantation | 1 | 0 | 1 |
| Relapsed | 0 | 0 | 0 |
| Death | 6 | 5 | 1 |

IST, immunosuppressive therapy; EPAG, eltrombopag; rATG, rabbit antithymocyte immunoglobulin; pALG. pig antilymphocyte immunoglobulin; CsA, cyclosporine A.
