# Supplementary figures and images for: Matched related transplantation versus immunosuppressive therapy plus eltrombopag for first-line treatment of severe aplastic anemia: a multicenter, prospective study
Source: J Hematol Oncol. 2022 Aug 12;15:105. doi: 10.1186/s13045-022-01324-1 (PMC9373485; doi:10.1186/s13045-022-01324-1)

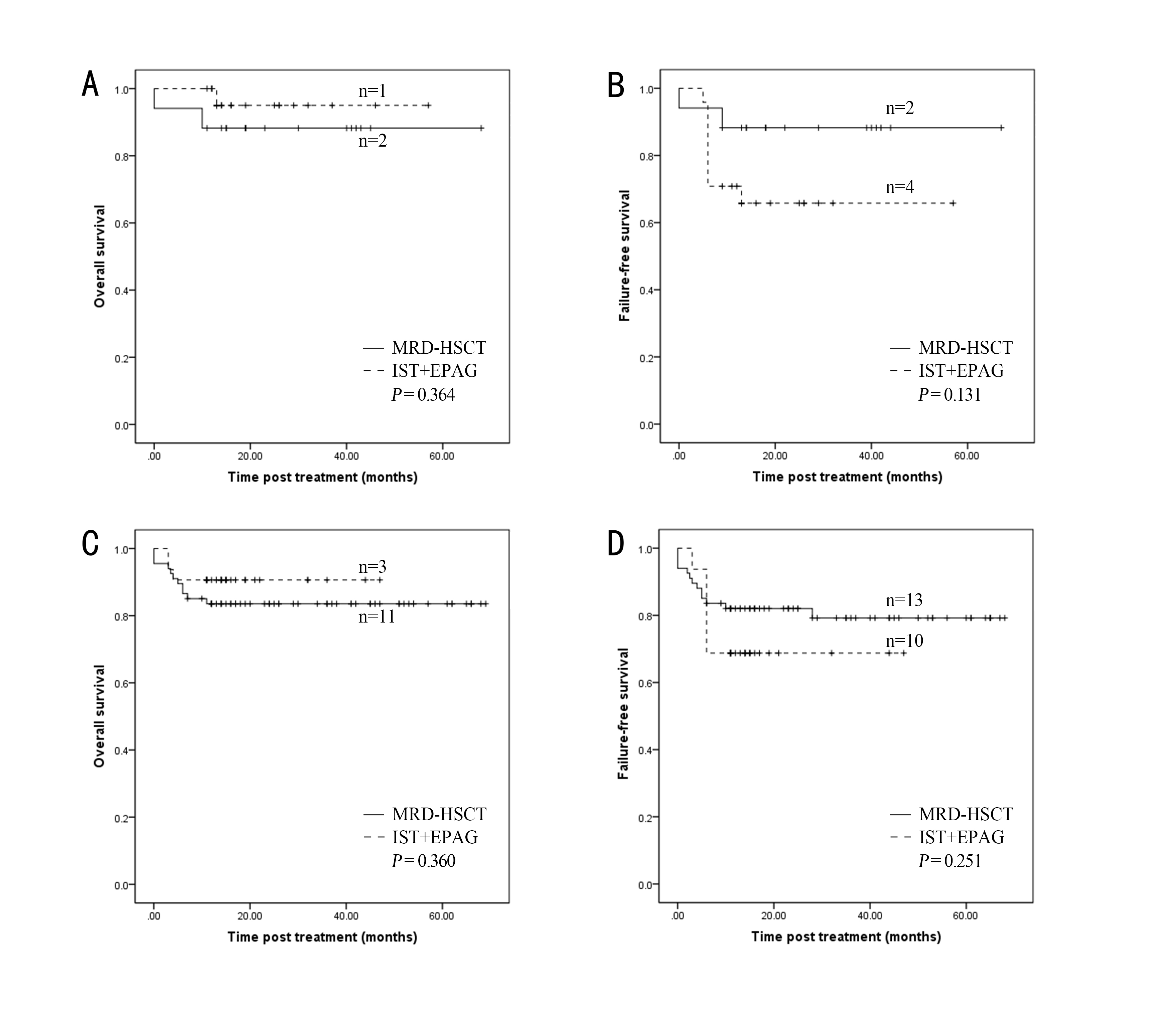

Supplement: Supplementary file 3 — Additional file 3: Fig. S1. Survival after treatment with MRD-HSCT or IST + EPAG for patients with further age stratification. (A) OS between MRD-HSCT and IST + EPAG subgroups for patients with aged < 20 years. (B) FFS between MRD-HSCT and IST + EPAG subgroups for patients age < 20 years. (C) OS between MRD-HSCT and IST + EPAG subgroups for patients with age 20–39 years. (D) FFS between MRD-HSCT and IST + EPAG subgroups for patients with age 20–39 years. [file 13045_2022_1324_MOESM3_ESM.jpg]
