# Supplementary material for: Matched related transplantation versus immunosuppressive therapy plus eltrombopag for first-line treatment of severe aplastic anemia: a multicenter, prospective study
Source: J Hematol Oncol. 2022 Aug 12;15:105. doi: 10.1186/s13045-022-01324-1 (PMC9373485; doi:10.1186/s13045-022-01324-1)
Supplement: Supplementary file 4 — Additional file 4: Table S2. Multivariate analysis of factors associated with favorable outcomes. [file 13045_2022_1324_MOESM4_ESM.doc]

**Table S 2. Multivariate analysis of factors associated with favorable outcomes**

| **Outcomes** | **Hazard ratio (95% CI)** | ***P*** |
| --- | --- | --- |
| Normal blood routine 6-month after treatment |  |  |
| Group (MRD-HSCT vs. IST+EPAG) | 0.293 (0.183–0.471) | **< 0.001** |
| Patient age (< 40 vs. ≥ 40 yr) | 0.757 (0.476–1.1206) | 0.241 |
| Disease status (SAA vs. vSAA) | 0.991 (0.652–1.507) | 0.968 |
| Median time from diagnosis to treatment (< 4 vs ≥ 4 mth) | 0.740 (0.481–1.141) | 0.173 |
| Overall survival |  |  |
| Group (MRD-HSCT vs. IST+EPAG) | 0.597 (0.259–1.378) | 0.227 |
| Patient age (< 40 vs. ≥ 40 yr) | 1.311 (0.574–2.998) | 0.521 |
| Disease status (SAA vs. vSAA) | 1.262 (0.544–2.932) | 0.588 |
| Median time from diagnosis to treatment (< 4 vs ≥ 4 mth) | 1.747 (0.774–3.943) | 0.179 |
| Failure-free survival |  |  |
| Group (MRD-HSCT vs. IST+EPAG) | 2.779 (1.531–5.045) | **0.001** |
| Patient age (< 40 vs. ≥ 40 yr) | 1.095 (0.632–1.897) | 0.747 |
| Disease status (SAA vs. vSAA) | 1.055 (0.604–1.841) | 0.851 |
| Median time from diagnosis to treatment (**<** 4 vs ≥ 4 mth) | 2.071 (1.205–3.561) | **0.008** |

CI, confidence interval; vs., versus; MRD-HSCT, matched related donor hematopoietic stem cell transplantation; IST, immunosuppressive therapy; EPAG, eltrombopag; SAA: severe aplastic anemia; vSAA: very SAA.
